# High prevalence of infections in non-COVID-19 patients admitted to the Emergency Department with severe lymphopenia

**DOI:** 10.1186/s12879-022-07295-5

**Published:** 2022-03-26

**Authors:** Arthur Baïsse, Thomas Daix, Ana Catalina Hernandez Padilla, Robin Jeannet, Olivier Barraud, François Dalmay, Bruno François, Philippe Vignon, Thomas Lafon

**Affiliations:** 1 Emergency Department, Limoges University Hospital Center, 2 avenue Martin Luther King, 87042 Limoges, France; 2 Inserm CIC 1435, Limoges University Hospital Center, 87042 Limoges, France; 3 Medical-Surgical Intensive Care Unit, Limoges University Hospital Center, 87042 Limoges, France; 4 Inserm UMR 1092, University of Limoges, 87042 Limoges, France; 5 CNRS UMR 7276 / INSERM U 1262, University of Limoges, Limoges, France; 6 Microbiology Department, Limoges University Hospital Center, 87042 Limoges, France; 7 Inserm UMR 1094, University of Limoges, 87042 Limoges, France

**Keywords:** Lymphopenia, Infection, Sepsis, Emergency, Biomarker

## Abstract

**Background:**

In the Emergency Department (ED), early and accurate recognition of infection is crucial to prompt antibiotic therapy but the initial presentation of patients is variable and poorly characterized. Lymphopenia is commonly associated with bacteraemia and poor outcome in intensive care unit patients. The objective of this retrospective study was to assess the prevalence of community-acquired infection in a cohort of unselected patients admitted to the ED with undifferentiated symptoms and severe lymphopenia.

**Methods:**

This is a retrospective single-center study conducted over a 1 year-period before the COVID-19 pandemic. Consecutive adult patients admitted to the ED with severe lymphopenia (lymphocyte count < 0.5 G/L) were studied. Patients with hematological or oncological diseases, HIV infection, hepato-cellular deficiency, immunosuppression, or patients over 85 years old were excluded. Diagnoses of infection were validated by an independent adjudication committee. The association between various parameters and infection was assessed using a multivariate logistic regression analysis.

**Results:**

Of 953 patients admitted to the ED with severe lymphopenia, 245 were studied (148 men; mean age: 63 ± 19 years). Infection was confirmed in 159 patients (65%) (bacterial: 60%, viral: 30%, other: 10%). Only 61 patients (25%) were referred to the ED for a suspected infection. In the univariate analysis, SIRS criteria (OR: 5.39; 95%CI: 3.04–9.70; *p* < 0.001) and temperature ≥ 38.3 °C (OR: 10.95; 95%CI: 5.39–22.26; *p* < 0.001) were strongly associate with infection. In the multivariate analysis, only SIRS criteria (OR: 2.4; 95%CI: 1.48–3.9; *p* < 0.01) and fever (OR: 3.35; 95%CI: 1.26–8.93; *p* = 0.016) were independently associated with infection.

**Conclusions:**

The prevalence of underlying infection is high in patients admitted to the ED with lymphopenia, irrespective of the reason for admission. Whether lymphopenia could constitute a valuable marker of underlying infection in this clinical setting remains to be confirmed prospectively in larger cohorts.

*Trial registration:* No registration required as this is a retrospective study.

**Supplementary Information:**

The online version contains supplementary material available at 10.1186/s12879-022-07295-5.

## Background

Infection is one of the most frequent reasons for admission to the Emergency Department (ED), with an overall mortality which still approximates 15% [1]. Early and accurate recognition of infections is therefore crucial to improve prognosis [2, 3]. Diagnosis of infection remains challenging since its clinical presentation is highly variable, currently available biological markers lack specificity and conventional microbiology typically requires 48–72 h to provide definite bacterial documentation [4, 5]. Accordingly, an additional yet simple biological marker would be of clinical value to help the front-line emergency physician efficiently screen patients for a potential underlying infection when presenting to the ED with undifferentiated symptoms.

Infection leads to an immune response associating both an excessive inflammation and immunosuppression [6]. Both the timing and magnitude of this response varies considerably and thus makes its identification difficult. Immune cells including granular, monocytic and lymphocytic lineages are involved [7]. Lymphopenia results from apoptosis which involves especially CD4 + cells [8]. Severe lymphopenia (lymphocyte count < 0.5 G/L) has been described as a prognostic marker in septic patients admitted to the Intensive Care Unit (ICU) and to the ED [9]. In addition, lymphopenia has been shown to have a higher diagnostic value than traditional biomarkers in predicting bacteremia in ED patients [10, 11]. Nevertheless, with the exception of the new-onset COVID-19 [12], lymphopenia as a potential biological marker to early search for an underlying infection in the ED has yet been scarcely studied.

Accordingly, the objective of this retrospective study was to assess the prevalence of community-acquired infection in a cohort of unselected patients admitted to the ED with undifferentiated symptoms and severe lymphopenia before the COVID-19 pandemic.

## Methods

We conducted a retrospective, descriptive, single-center study between January and December 2017 in the ED of a French University Hospital. All patients over 18 years who presented with severe lymphopenia (lymphocyte count ≤ 0.5 G/L) on ED admission (first blood cell count) were studied [9]. Patients with chronic hematological or oncological diseases, immunosuppression (Human Immunodeficiency Virus, transplant, treatment with steroids at any dose or immunotherapy for more than three months), hepatocellular insufficiency defined as any chronic diseases associated with an impairment of hepatocyte functions [13], and elderly patients (age > 85 years considered as having physiological immunosenescence) were excluded [14–16].

Diagnoses of community-acquired infection were validated by an independent adjudication committee (composed of an experienced emergency physician and intensivist), according to clinical, biological and microbiological data [17]. Diagnosis of infection was based on the presence of two of the three following pre-defined criteria: (i) the presence of a potential source of infection, (ii) microbiology data, and (iii) patient outcome under antibiotic therapy.

Demographic data, Systemic Inflammatory Response Syndrome (SIRS) criteria (Respiratory rate > 20 breaths /min, WBC count > 12 G/L or < 4 G/L, temperature > 38.3 °C or < 36 °C, heart rate > 90 bpm) [18], quick Sequential Organ Failure Assessment (qSOFA) (change in mental status, respiratory rate ≥ 22 breaths/min, systolic blood pressure ≤ 100 mmHg) [19], site of infection, microbiology, and biological parameters (leukocyte count, lymphocyte count, CRP, lactates) were collected in each patient. Comorbidities including diabetes, chronic renal failure (defined as creatinine clearance ≤ 30 mL/min), chronic respiratory failure (defined as long-term oxygen therapy), severe heart failure (defined by a left ventricular ejection fraction ≤ 30%), peripheral vascular diseases (obliterant vascular disease and stroke) were also recorded.

Lymphocyte and leukocyte counts were performed using an ADVIA 2120i meter connected to the GLIMS network via a PGP network. This counting method has a margin of error of ± 0.2–0.3 G/L for lymphocyte values lower than 4.0 G/L.

Descriptive statistics, including mean (standard deviation) and frequency distributions were used to describe the cohort. Normality of continuous variables was tested using Shapiro–Wilk test. Comparison between patients with and without infection was performed using the Chi 2 or Fischer test for categorical variables and the Students t-test (equal variances) or nonparametric Mann–Whitney U test for continuous data with non-normality. The clinical and laboratory criteria associated with the diagnosis of infection were evaluated by univariate and multivariate analysis using a logical regression model. The criteria retained in the multivariate analysis were those significant in univariate analysis as well as those for which the p-value was < 0.2. A *p-*value smaller than 0.05 using a two-sided test was considered statistically significant. To take into account the potential effects of seasons on the prevalence of infectious diseases, we decided to conduct a one-year observational study, which would allow enrolling a representative sample size.

## Results

**Fig. 1:**
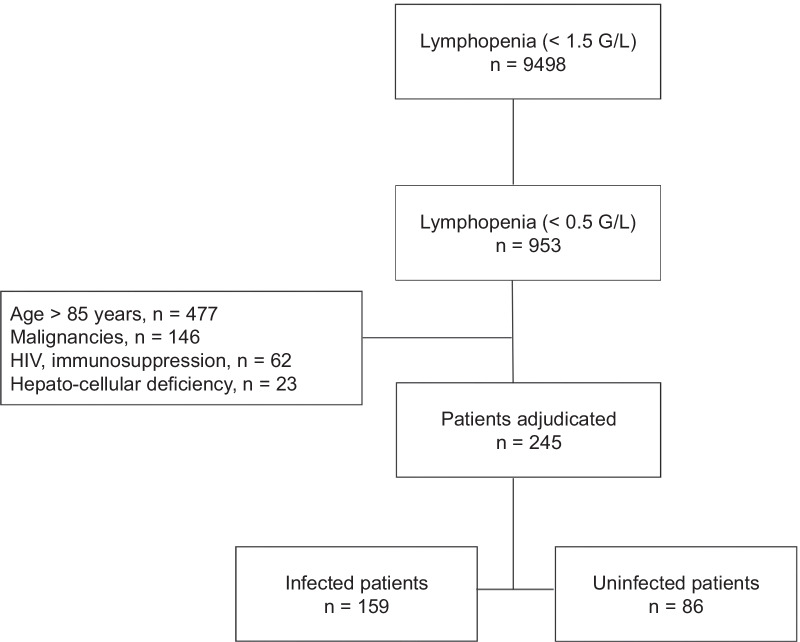
Flowchart of the study

During the study period, 21,914 of 43,258 patients admitted to the ED underwent a blood cells count, and 9498 of them presented with a lymphopenia < 1.5 G/L, including 953 patients with severe lymphopenia (Fig. 1). Among them, 708 patients were excluded for a chronic oncological/hematological disease (n = 146), immunosuppression (n = 62), hepatocellular failure (n = 23), or an age > 85 years (n = 477). Finally, the analysis was performed in 245 patients (mean age: 63 ± 19 years; 148 men [60%]). Most common comorbidities included diabetes (23%), severe heart failure (24.5%) and chronic peripheral vascular disease (15%). Only 62 patients (26%) were referred to the ED for a suspected infection, including 21 patients (9%) for unexplained fever (Table 1).

**Table 1:** Baseline clinical and biological characteristics of the study population (n = 245)

	Infected patients	Non-infected patients	p-value
	n = 159 (%)	n = 86 (%)	
Age (year)	60 ± 20	69 ± 14	0.003
Sex (male)	95 (60)	53 (62)	0.61
Co-morbidities			
Diabetes	34 (21)	23 (27)	0.34
Chronic renal insufficiency	10 (6)	8 (9)	0.39
Chronic respiratory insufficiency	5 (3)	4 (5)	0.55
Chronic cardiac insufficiency	34 (21)	26 (30)	0.13
Peripheral arterial disease	10 (6)	5 (6)	0.90
Cerebrovascular disease	11 (7)	10 (12)	0.21
Suspected infectious reason for admission	61 (38)	1 (1)	< 0.001
Infections diagnosed in ED	131 (82)	5 (6)	< 0.001
SIRS criteria ≥ 2	114 (72)	25 (29)	< 0.001
Heart beat > 90 / mn	110 (69)	37 (43)	< 0.001
Respiratory rate > 20 / mn	84 (53)	14 (16)	< 0.01
Temperature > 38,3 °C or < 36 °C	98 (62)	11 (13)	< 0.001
Leukocytes > 12 G/L or < 4 G/L	55 (35)	27 (31)	0.82
Biology			
Leukocytes (G/L)	10.8 ± 5.5	10.9 ± 5.9	0.98
Lymphocytes (G/L)	0.35 ± 0.11	0.37 ± 0.11	0.12
Neutrophils (G/L)	9.89 ± 5.3	9.95 ± 5.7	0.98
Platelets (10^3^/µL)	192 ± 81	221 ± 82	0.01
CRP (mg/L)	109 ± 133	46 ± 65	< 0.001
Lactates (mmol/L)	2.3 ± 1.6	2.5 ± 1.7	0.47

*SIRS* systemic inflammatory response syndrome, *CRP* C-reactive protein

Numbers in parentheses denote percentages

**Fig. 2:**
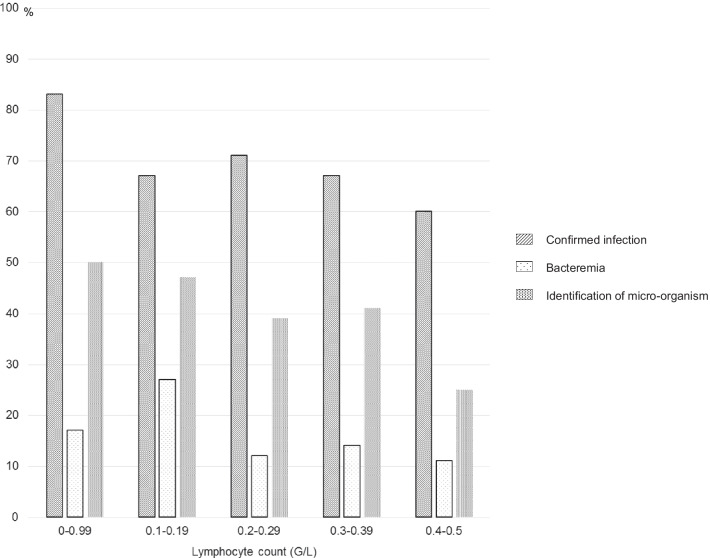
Prevalence of infections, bacteremia and isolated micro-organisms according to lymphocytes count

The adjudication committee confirmed the diagnosis of infection in 159 patients (65%) (95 men; mean age: 60 ± 20 years). In this subset of patients, infection was not diagnosed in the ED, but during the subsequent hospitalization in 18% of cases (Table 1). Finally, 96 patients (61%) were diagnosed with a bacterial infection. Infectious sites were predominantly pulmonary (28%), digestive (23%), urinary (22%) and cutaneous (8%). A micro-organism was identified in 85 patients (53%). Among bacterial infections, the most frequently isolated micro-organisms were gram-negative bacilli (25%). Viral infection was documented in 19 patients (40%) (Additional file 1: Table S1). Overall, 114 patients (72%) presented at least two SIRS criteria and 46 patients (29.5%) had a qSOFA score ≥ 2 points on ED admission. There was no statistically significant difference of lymphocytes count between patients with bacterial and viral infections (0.350 ± 0.110 vs 0.351 ± 0.120: *p* = *0.893*). Profound lymphopenia (lymphocyte count < 0.1 G/L) was associated with a even higher prevalence of infection (83%) and bacteremia (50%), the difference failing to reach statistical significance (*p* = 0.42 and 0.69, respectively). The rate of identification of micro-organisms tended to increase with the severity of lymphopenia, the difference being not statistically significant (Fig. 2).

CRP level was measured in 240 patients (98%) and was significantly higher in infected patients when compared to their counterparts (109 ± 133 vs 46 ± 65 mg/L: *p* < 0.001). In contrast, leukocytes count and lactate were not statistically different between groups (10.8 ± 5.5 vs 10.9 ± 5.9 G/L: *p* = *0.98*, and 2.3 ± 1.6 vs 2.5 ± 1.7 mmol/L: *p* = *0.38*, respectively) (Table 1). In univariate analysis, SIRS criteria (OR: 5.39; 95%CI: 3.04–9.70; *p* < *0.001*) and body temperature ≥ 38.3 °C (OR: 10.95; 95%CI: 5.39–22.26; *p* < *0.001*) were strongly associated with infection (Table 2). In the multivariate analysis, SIRS criteria (OR: 2.4; 95%CI: 1.48–3.9; *p* = *0.0004*) and fever (OR: 3.35; 95%CI: 1.26–8.93; *p* = *0.016*) were identified as independent variables associated with the diagnosis of underlying infection (Table 3).

**Table 2:** Univariate analysis

	OR	*p*	95% CI
Age ≥ 75 years	0.61	0.09	0.35 – 1.07
Sex (male)	0.92	0.79	0.54 – 1.56
SIRS criteria ≥ 2	5.39	< 0.001	3.04 – 9.70
Temperature ≥ 38.3 °C	10.95	< 0.001	5.39 – 22.26
Heart beat ≥ 90 / mn	2.97	< 0.01	1.73 – 5.12
Respiratory rate ≥ 20 / mn	3.29	< 0.01	1.54 – 7.03
Leukocytes > 12 or < 4 G/L	1.15	0.67	0.67 – 2.02
Lactates > 2 mmol/L	0.84	0.58	0.50 – 1.49
CRP ≥ 50 (mg/L)	2.03	0.01	1.19 – 3.46
Platelets < 150 10^3^/µL	2.29	0.02	1.19 – 4.67

*SIRS* systemic inflammatory response syndrome, *CRP* C-reactive protein, *OR* odds ratio, *CI* confidence intervals

**Table 3:** Multivariate analysis using logical regression model

	OR	*p*	95% CI
SIRS criteria ≥ 2	2.4	< 0.01	1.48—3.9
Temperature ≥ 38.3 °C	3.35	0.016	1.26 – 8.93

*SIRS* systemic inflammatory response syndrome, *OR* odds ratio, *CI* confidence intervals

## Discussion

The present study conducted before the COVID-19 pandemic showed that the prevalence of infection reached 65% in patients who present to the ED with severe lymphopenia, irrespective of the reason for admission. Infection rate reached 83% in patients with profound lymphopenia and bacterial infections were predominant (61%). SIRS criteria and especially fever still have been found to be independently related to infection.

According to the Surviving Sepsis Campaign, early and accurate recognition of infections is crucial to improve the prognosis of sepsis, particularly through the prompt initiation of adapted antimicrobial therapy and of fluid resuscitation [3]. Diagnosis of infection in the ED is based on heterogeneous, non-specific clinical and biological signs, and therefore remains challenging in the clinical setting of unpredictable workload and associated critical care [4]. Minderhoud et al. [20] described only 1/3 of bacterial confirmed infection an 1/3 of suspected infection from a cohort of 269 patients in ED with suspected sepsis. Moreover, Heffner et al. [21] found that 50% of patients identified and treated for sepsis in the emergency department had negative culture results. In the present study, only 38% of patients with secondarily identified infection were initially referred to the ED for a suspected infection. This reflects the complexity of early and accurate recognition of infection which diagnosis is often only presumptive [22]. Recently, Shappell et al. [23] reported that one third of patients empirically treated with broad-spectrum antibiotics in the ED are ultimately diagnosed with non-infectious or viral conditions. Klein Klouwenberg et al. [24] reported a post-hoc plausibility of infection in 43% of ICU patients who were not initially considered as infected. Accordingly, the availability of a simple yet robust biological marker strongly associated with infection would be of clinical value in the ED settings, since microbiology testing is not adequately suited to provide information within a suitable timeframe [25].

The use of conventional biomarkers (i.e., leukocyte and neutrophil counts, CRP) has shown limited predictive ability with low specificity for the diagnosis of infection [26]. In keeping with these results, markers of inflammation failed to be independently associated with the presence of an underlying infection in our study population. In contrast, infection was the cause of lymphopenia in 65% of cases, and in up to 83% of cases when it was profound (lymphocyte count < 0.1 G/L), irrespective of the reason for ED admission. Lymphocyte count is easy to obtain and constitutes a simple yet robust biological parameter with a higher diagnostic performance than other biomarkers traditionally used, such as CRP level, white blood cell and neutrophil counts [10].

Severe lymphopenia has been considered as a prognosis marker at the late phase of sepsis, especially in the ICU [9] and was also considered as a biomarker of bacteremia in various age groups [14, 27]. The mechanisms responsible for lymphopenia during infections involve margination of lymphocytes and marked accelerated apoptosis, a prominent feature of sepsis [28]. These results are compatible with the rapid decline in blood lymphocyte count occurring in animal [29] and human models of sepsis [30, 31]. Accordingly, the use of the first lymphocyte count on ED admission appears clinically relevant. Although the relation between lymphopenia and infection has been previously described, this association has yet been poorly studied in the ED settings [32]. Our results suggest that lymphopenia should prompt the front-line intensivist to search for an underlying infection due to its large prevalence in this clinical setting.

Although following the new Sepsis-3 definition the use of both the SOFA and qSOFA scores allows anticipating subsequent patient’s course [19], it fails allowing an accurate diagnosis of infection or sepsis. In the patients with severe lymphopenia, SIRS criteria—especially fever—appeared independently associated with the identification of an underlying infection, even though 28% of our patients had no SIRS criteria and 38% were not febrile. This diagnostic capability is in keeping with the results of previous studies which reported a greater diagnostic performance of SIRS criteria than the qSOFA score [33]. Although the SIRS criteria include the presence of a leukocytosis (> 12 G/L) or of a leukopenia (< 4 G/L), it fails using the leukocyte subpopulations. Eosinopenia has also been shown to be predictive of sepsis in the ED settings, with a higher performance than other markers such as CRP and PCT [34]. Finally, severe functional deficits of monocytes have been described in septic patients and contribute to their immunosuppressive state [35].

In the study population, underlying infections were predominantly of bacterial origin and gram-negative bacilli (especially *Escherichia Coli* and *Klebsiella pneumoniae*) were the most frequently isolated micro-organisms, a well-known microbiological epidemiology encountered in the ED setting [20, 27]. On clinical grounds, early distinction between viral and bacterial infections is challenging when only based on clinical and routinely available biological findings. Lymphopenia is known to be more pronounced in with the presence of a bacterial infection than in patients presenting with a viral infection [36]. Nevertheless, it failed discriminating patients with bacterial and viral infections in our cohort. In addition, lymphopenia has been shown to be a valuable diagnostic and prognostic marker of COVID-19 disease [37]. Accordingly, our data cannot be extrapolated to suspect a bacterial coinfection in patients presenting to the ED with COVID-19 pneumonia.

The diagnostic performance of severe lymphopenia could not be fully assessed because of the retrospective design of this observational study precluding to constitute a control group without lymphopenia. Similarly, confounding factors predisposing to chronic or iatrogenic lymphopenia (e.g., malnutrition) could not be taken into account [14]. Since we analyzed only patients with severe lymphopenia on ED admission, the potential diagnostic value of lymphopenia developing within the first hours of admission has not been assessed [31]. Nevertheless, underlying infections were diagnosed based on an independent adjudication committee which was blinded to the lymphocytes count and this pragmatic study has been performed in the challenging clinical setting of patients presenting to the ED with undifferentiated symptoms. Since procalcitonin level is not systematically determined in our ED, we were not able to use this biological marker in the present study. Finally, since patients with COVID-19 frequently present with lymphopenia, the presented results need to be challenged prospectively in the current era of COVID-19 pandemic.

## Conclusion

The prevalence of infections was high in patients admitted to the ED with severe lymphopenia before the COVID-19 pandemic, and appeared even higher in the presence of profound lymphopenia. Irrespective of the reason for ED admission and clinical symptoms, lymphopenia associated with SIRS criteria appeared to be strongly associated with an underlying infection, most frequently of bacterial origin. Accordingly, this simple biological marker, which can be early and easily obtained in the ED, should prompt the emergency physician to search for SIRS criteria and underlying infection.

## Supplementary Information

**Additional file 1: Table S1. **Additional table showing the micro-organisms isolated in patients with confirmed infections.

## Data Availability

All data generated or analyzed during this study are included in this published article and its supplementary information files.

## Abbreviations

COVID-19: Coronavirus disease 2019
CRP: C-reactive protein
PCT: Procalcitonin
HIV: Human immunodeficiency virus
SIRS: Systemic inflammatory response syndrome
SOFA: Sequential organ failure assessment
