# Supplementary material for: High prevalence of infections in non-COVID-19 patients admitted to the Emergency Department with severe lymphopenia
Source: BMC Infect Dis. 2022 Mar 26;22:295. doi: 10.1186/s12879-022-07295-5 (PMC8960225; doi:10.1186/s12879-022-07295-5)
Supplement: Supplementary file 1 — Additional file 1: Table S1. Additional table showing the micro-organisms isolated in patients with confirmed infections. [file 12879_2022_7295_MOESM1_ESM.docx]

**Additional file 1**

Table S1. Micro-organisms isolated in patients with confirmed infections.

|  | Organisms isolated |
| --- | --- |
|  | n (%) |
| Bacteria | 61 (38) |
| Gram negative bacilli |  |
| *Escherichia Coli* | 21 (13) |
| *Klebsiella spp.* | 5 (3) |
| *Morganella Morganii* | 2 (1) |
| *Campylobacter* | 3 (2) |
| Proteus mirabilis | 3 (2) |
| *Citrobacter spp.* | 2 (1) |
| Others | 3 (2) |
| Gram positive cocci |  |
| Staphylococcus spp. | 10 (6) |
| Streptococcus spp. | 10 (6) |
| Others* | 2 (1) |
| Viruses | 19 (12) |
| Influenzae | 13 (8) |
| Measles | 3 (2) |
| Syncytial Respiratory Virus | 2 (1) |
| Varicella Zoster Virus | 1 (1) |
| Parasites | 5 (3) |
| Plasmodium falciparum | 5 (3) |
| Non identified | 74 (47) |

** Eggerthella, Veillonella*
